# Mechanical Properties and In Vitro Degradation of Sputtered Biodegradable Fe-Au Foils

**DOI:** 10.3390/ma9110928

**Published:** 2016-11-15

**Authors:** Till Jurgeleit, Eckhard Quandt, Christiane Zamponi

**Affiliations:** Chair for Inorganic Functional Materials, Institute for Materials Science, Faculty of Engineering, University of Kiel, Kiel 24143, Germany; eq@tf.uni-kiel.de (E.Q.); cz@tf.uni-kiel.de (C.Z.)

**Keywords:** biodegradable, iron, mechanical properties, magnetron sputtering

## Abstract

Iron-based materials proved being a viable candidate material for biodegradable implants. Magnetron sputtering combined with UV-lithography offers the possibility to fabricate structured, freestanding foils of iron-based alloys and even composites with non-solvable elements. In order to accelerate the degradation speed and enhance the mechanical properties, the technique was used to fabricate Fe-Au multilayer foils. The foils were annealed after the deposition to form a homogeneous microstructure with fine Au precipitates. The characterization of the mechanical properties was done by uniaxial tensile tests. The degradation behavior was analyzed by electrochemical tests and immersion tests under in vitro conditions. Due to the noble Au precipitates it was possible to achieve high tensile strengths between 550 and 800 MPa depending on the Au content and heat treatment. Furthermore, the Fe-Au foils showed a significantly accelerated corrosion compared to pure iron samples. The high mechanical strength is close to the properties of SS316L steel. In combination with the accelerated degradation rate, sputtered Fe-Au foils showed promising properties for use as iron-based, biodegradable implants.

## 1. Introduction

The treatment of cardiovascular diseases with permanent implants such as stents is state of the art. However, it was shown that after a certain healing period of three to 12 months [1,2], the vessel has remodeled itself. Afterwards, besides lacking a purpose for the healing, the implant acts as a foreign body which can lead to complications such as late stent restenosis and chronic inflammation reactions. One promising approach is to solve this problem with the use of non-permanent biodegradable implants. Biodegradable implants can consist of all material classes (metals, polymers and ceramics). One of the most important properties is the biocompatibility of the material as well as its degradation products. Furthermore, the material has to keep its mechanical integrity during the healing period and degrade fast enough not to cause the mentioned complications. Therefore, the degradation should be neither too fast nor too slow. The most prominent examples for biodegradable metals are magnesium and iron. While magnesium degrades rather fast and with hydrogen evolution, the in vivo degradation of pure iron was found to be slow but without significant hydrogen evolution [3,4,5,6] and thus reduces the risk of complications due to subcutaneous gas accumulations. Therefore, the challenge for iron-based materials is to either accelerate the corrosion rate or improve the mechanical properties insofar as developing thinner structures, and thus less material is sufficient to resist the load acting on the implant. It was shown by Peuster et al. [7,8] that biodegradable iron stents can be implanted into New Zealand rabbits and miniature pigs without observing any complications such as inflammation reactions, neointimal proliferation, or thrombotic events.

In the past different approaches were followed to fulfill these challenges. Moravej et al. [9] showed that due to its microstructure, electroformed iron (E-Fe) shows a higher in vitro corrosion rate compared to cast iron. 

In order to enhance the mechanical properties and the degradation performance, also the implementation of noble Pd [10,11], Pt [11], Au and Ag [12,13] were investigated. In the work [13] by Huang et al., they implemented the noble precipitates by using powder metallurgy and spark plasma sintering. These precipitates act as micro-galvanic elements and thus accelerate the degradation rate. Furthermore, it was found that the formation of precipitates increases the compressive strength of the material. Additionally, they demonstrated a sufficient in vitro biocompatibility of Fe-Ag and Fe-Au composites. Due to the poor solubility or even insolubility of Au and Ag in Fe, those Fe composites cannot be produced by conventional cast techniques. Therefore, alternative fabrication techniques are required. In recent years, several studies [14,15,16,17,18,19,20] showed that magnetron sputtering technology in combination with structuring techniques used in micro-system technology is suitable to achieve micro-patterned metallic NiTi and Mg devices. In a previous work by the authors [21] it was presented, that the technique is even suitable for the fabrication of patterned pure Fe foils. The devices showed a high strength and a comparable degradation behavior in comparison with pure cast iron [9]. In order to accelerate the degradation speed and further enhance the mechanical properties, in another work [12] the authors investigated the microstructure of sputtered Fe-Au foils. It was shown that by annealing Fe-Au multilayer foils, it is possible to achieve a homogeneous microstructure where the Au exists in the form of finely distributed precipitates in the Fe matrix. In this study, the focus was put on the investigation of the tensile properties and the degradation behavior of such already lithographically micro structured Fe-Au foils, in order to understand how these precipitates influence the material properties. For this purpose, structured Fe-Au foils of various compositions were fabricated and characterized.

## 2. Experimental Section 

### 2.1. Preparation of Samples 

All metal films were deposited in a CS730S cluster magnetron sputtering machine (VON ARDENNE, Dresden, Germany). As working gas Ar was used. For the corrosion measurements quadratic foils of 15 mm edge length and 10 µm thickness were fabricated. A “dog-bone” shaped design with a strut width of 0.5 mm, 7 mm strut length and a homogeneous thickness of 30 µm was used. For the patterning of the foils 4 inch Si wafers were structured by performing UV-lithography and galvanic Cu deposition [16]. The sputtering parameters are given in Table 1. 

Three different multilayer systems in terms of the Au layer thickness were produced. While the thickness of the Fe layers were kept constant 1 µm for all samples, the Au layer thickness was varied in order to fabricate samples with different amounts of Au. The samples are henceforth referred as (FeAu0.3), (FeAu1.0) and (FeAu2.5). The number used in the nomenclature of the samples refers to the average Au content in at %, measured at 10 different spots by EDX (energy dispersive X-ray spectroscopy). All samples were fabricated as reported previously [12]. In order to homogenize the samples they were annealed at 800 °C for two hours. Additional tensile test samples were annealed at 600 °C for two hours, in order to evaluate the mechanical behavior of samples with partially intact multilayer structure. 

### 2.2. Corrosion Measurements

For the determination of the corrosion behavior electrochemical linear polarization measurements and immersion tests were performed. The corrosion tests were performed using stirred Hank’s buffered salt solution (H1387 Sigma Aldrich, Taufkirchen, Germany), modified with sodium bicarbonate (0.35 g/L). The temperature of the solution was held constant at 37 ± 1 °C, while the pH was regulated by CO_2_ inlet to obtain a value of 7.4 ± 0.05. 

Since after the deposition the sample surface exhibited mirror finish quality (R_a_ = 14 ± 3 nm), no further polishing was necessary. Prior to testing the samples were rinsed with isopropanol and DI-water and fast dried afterwards. A VersaSTAT 3-300 potentiostat connected to a three-electrode cell was used for the electrochemical corrosion tests. The samples were mounted on the sample holder acting as the working electrode (WE) with an exposed area of 0.916 cm². As counter electrode (CE) a Pt- mesh and Pt-wire was used. An Ag/AgCl electrode in a 3 molar KCl solution acted as the reference electrode (RE). After determining the corrosion potential U_c_ in a 4000 s open circuit (OC) measurement the measurement of the I(U) curves was performed. Therefore, the WE was polarized from −400 to +400 mV around U_c_ with a potential shift rate of 1 mV/s. The Tafel extrapolation method was used to calculate the corrosion rate (CR) from the I(U) curves. For that reason the current was converted into the current density. By fitting the linear regimes of the logarithmically plotted j(U) curve and extrapolation to U_c_, the corrosion current density j_c_ was determined. The corrosion rate was calculated using Equation (1) which is based on Faraday’s law [22,23].

(1) $$\mathrm{CR}=\;\frac{j_{c}M}{n\rho F}$$

Here M is the molar mass of the corroding species, ρ the density, n the number of transferred elementary charges per reaction step and F Faraday constant. Under the assumption based on studies by Zhu et al. [24] on the degradation kinetics of pure-iron in physiological fluids, iron is anodic dissolved as followed $Fe-2e^{-}\to \;Fe^{2+}$, so that n = 2. With, M = 0.056 kg/mol, ρ = 7874 kg/m³ and a conversion factor of 31,536, the CR can be calculated in terms of mm/year. For the immersion tests the weight of the samples was determined using a high accuracy balance and afterwards different samples were immersed in 600 mL solution for two, four, six and 12 days. After immersion the corrosion products were carefully removed, the samples were rinsed in isopropanol and fast dried with nitrogen to prevent further oxidation. Afterwards the weight loss was determined. Four different samples of each type were measured. In order to evaluate the microstructure of the samples SEM surface images and EDX elemental mappings were prepared using a scanning electron microscope (Zeiss Ultra Plus, Oberkochen, Germany). Furthermore, sample cross sections were prepared by focused ion beam (FIB) using a Helios NanoLab 600 (FEI). The cross sections were investigated by scanning transmission electron microscopy (STEM) using a F30 G² -ST (Tecnai, FEI, Frankfurt, Germany).

### 2.3. Mechanical Properties

Tensile tests were performed for mechanical characterization using dog-bone-shaped samples with 0.5 mm strut width and 7 mm strut length. The uniaxial tensile tests were performed with a testing machine of the type BETA 5-5/6 × 10 (Messphysik, Fürstenfeld, Austria) with a special sample holder for thin samples. A straining rate of 0.4%/min was applied. For the fracture criterion a force reduction of 60% relative to the maximum applied force was used. For each type four samples were measured to assess the statistic reliability.

## 3. Results and Discussion

Freestanding pure Fe and Fe-Au multilayer samples were produced by magnetron sputtering. The samples were characterized in terms of their mechanical properties and corrosion behavior. The mechanical properties of the pure Fe samples and different multilayer systems were evaluated from the stress-strain curves measured before and after annealing at 800 °C. The results are shown in the following subsection.

### 3.1. Mechanical Properties 

**Figure 1:**
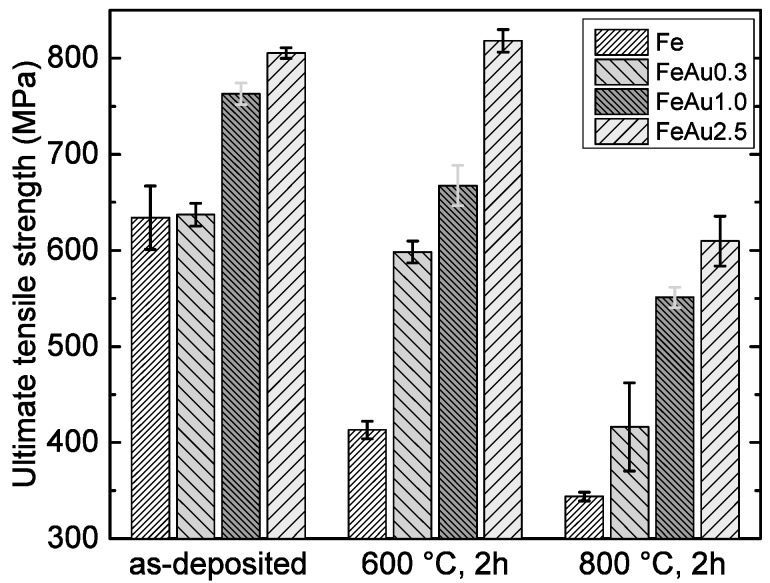
Ultimate tensile strength of the Fe, FeAu0.3, FeAu1.0 and FeAu2.5 samples, before and after different heat treatments.

**Figure 2:**
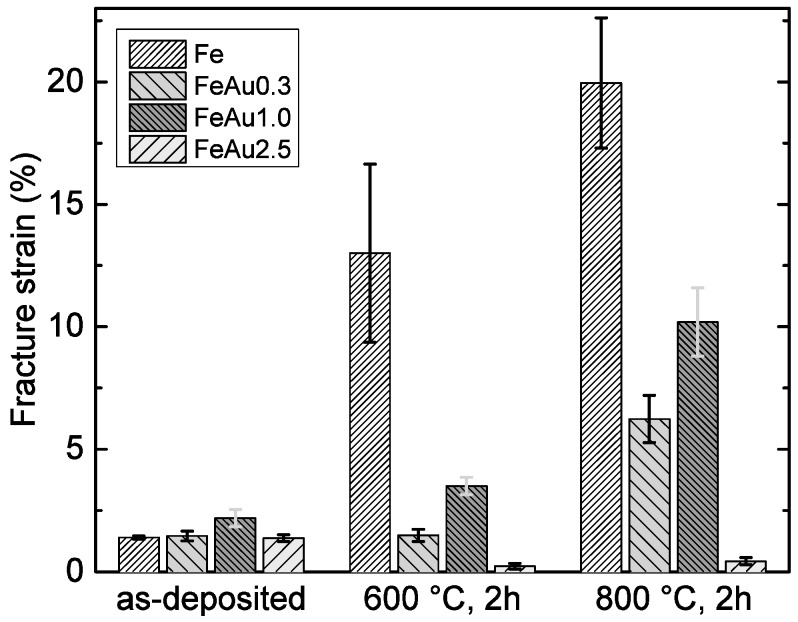
Fracture strain of the Fe, FeAu0.3, FeAu1.0 and FeAu2.5 samples, before and after different heat treatments.

From the stress-strain curves, the characteristic values of the ultimate tensile strength *R_m_* and the fracture strain were determined for all samples. For each type of sample, the mean values and standard deviations were calculated. The results are shown in Figure 1 and Figure 2. The high initial strength and the decrease of strength and increased ductility of pure Fe samples at higher annealing temperatures were discussed in a previous work [21]. It was found that the as-deposited foils show an energetically unfavorable, defect-rich, columnar and fine-grained structure which is characteristic for sputtered material. During annealing, secondary recrystallization was observed, leading to the reduction of defects and grain coarsening. Compared to the pure Fe samples, the Fe-Au samples show a higher strength and decreased fracture strain scaling with the Au content. While the increased strength scaling with the amount of gold is an expected behavior and is attributed to the size and amount of the Au precipitates, the behavior of the plastic deformation is less obvious. In general, it is expected that plasticity decreases, scaling with the size and the amount of the precipitates and thus the Au content, respectively. 

**Figure 3:**
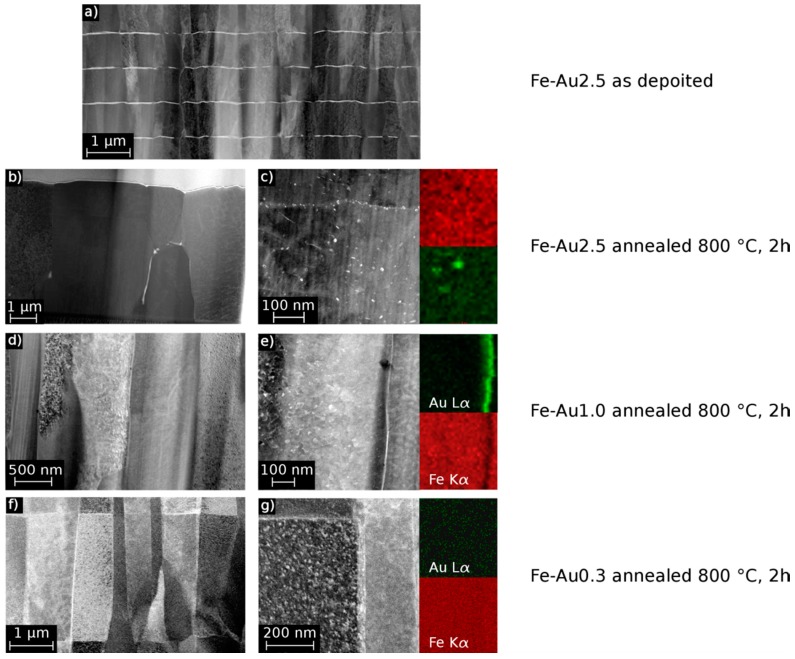
STEM images and EDX Fe-K-α and Au-L-α elemental mappings of Fe-Au sample cross-section, as deposited (**a**) and annealed at 800 °C for two hours (**b**–**g**). In the images Au precipitates appear bright due to the material contrast. (**a**) FeAu2.5 sample as deposited with intact multilayer structure; (**b**) FeAu2.5 sample annealed and (**c**) Fe-Au STEM/EDX mapping of Au precipitates in a grain and along a grain boundary; (**d**) FeAu1.0 sample annealed; the bright Au precipitates are barely visible along the grain boundaries but still resolvable in the EDX mapping (**e**). FeAu0.3 sample annealed (**f**), bright areas along the grain boundaries cannot be identified by EDX mapping (**g**).

However, the annealed FeAu0.3 samples show a significant lower elongation compared to the FeAu1.0 samples, while the FeAu2.5 samples show, as expected, the lowest value. A possible explanation for the unexpected behavior of the annealed FeAu0.3 samples can be found in the STEM images (Figure 3). While the elemental mappings of the FeAu2.5 and FeAu1.0 samples clearly show Au precipitates, in the elemental mapping of the FeAu0.3 sample no evidence of Au precipitates can be found. The Au signal is statistically distributed over the entire sample. According to the Fe-Au phase diagram, the solubility of Au in Fe at 800 °C is around 1 at % [25]. This suggests that due to the low Au content, the solubility during the annealing is high enough to dissolve the Au in the Fe matrix. With the cooling rate of 0.7 K/s, the diffusion in the sample decreases very fast. As a consequence, the gold atoms are trapped in the lattice, preferably at dislocations and grain boundaries. Due to the nominal Au content of 0.3 at %, low diffusion and homogeneous distribution during annealing, the critical nucleus size necessary for the formation of Au precipitates cannot be reached. Based on the mismatch of the different atomic radii (Fe = 124.1 pm, Au = 144.2 pm), the trapped Au atoms hinder the movement of dislocations and induce internal stresses which lead to a solid solution hardening and reduced plasticity. The decrease of strength at higher annealing temperatures is observed for all samples. The observed behavior is also attributed to the reduction of crystal defects and grain coarsening during annealing. This allows an easier dislocation movement and plastic deformation. It was shown in previous a work [12] that by annealing the multilayer structure (Figure 3a) of Fe-Au, the samples dissolve (Figure 3b–g). Afterwards, two kinds of precipitates exist, one in the grains and the second along grain boundaries (Figure 3b,c). These precipitates act as obstacles for the dislocation movement which enhance the strength while slightly decreasing the ductility of the material. The average grain size of the annealed samples (Table 2) might be an additional reason. All Fe-Au samples exhibit, independent of the Au content, a smaller grain size compared to the annealed pure iron. In agreement with the Hall-Petch relation [26,27] smaller grains will lead to a higher strength. However, since the strength clearly increases with the Au content and the different Fe-Au samples have more or less the same grain size, the precipitation hardening seems to have the major influence. From the results shown in Figure 1 and Figure 2, the FeAu1.0 samples show the best compromise between high strength and ductility. The observed tensile strength of the annealed FeAu1.0 (551 MPa) samples is much higher compared to pure cast iron [9] (205 MPa) and sputtered pure iron (343 MPa). The value even approaches the tensile strength of the SS316L alloy (580 MPa), which is the gold standard for cardiovascular stents [9]. The tensile strength is higher than desired values (300 MPa) by almost a factor of two [28] for biodegradable scaffolds. The elongation, however, comes close to the desired values (15%) [28] but should be further improved by varying the annealing parameters (temperature, time, cooling rate), layer thickness and sequence in order to optimize the size and distribution of the precipitates.

**Figure 4:**
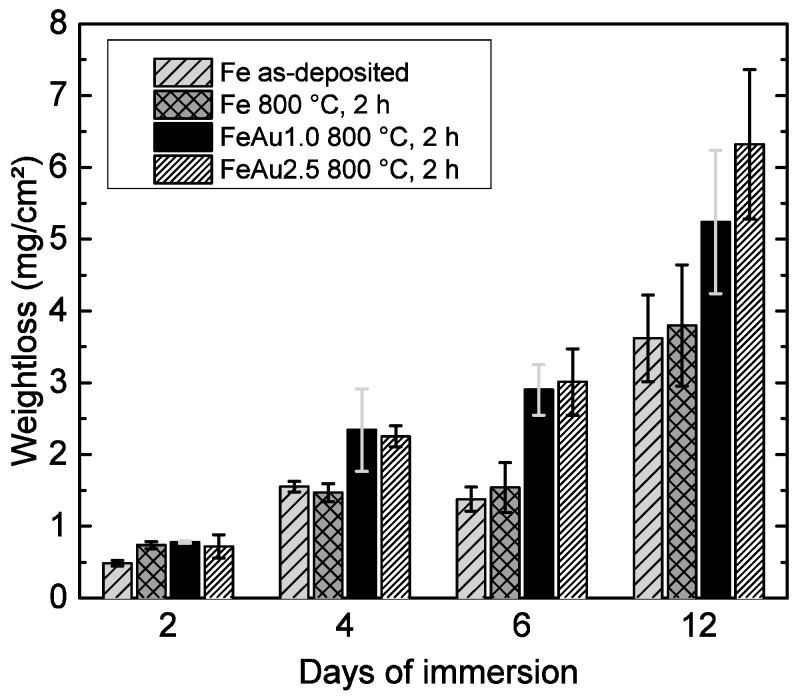
Weight loss of Fe, FeAu0.3, FeAu1.0 and FeAu2.5 samples, after immersion for different times in HBSS (pH 7.4; 37 °C).

**Figure 5:**
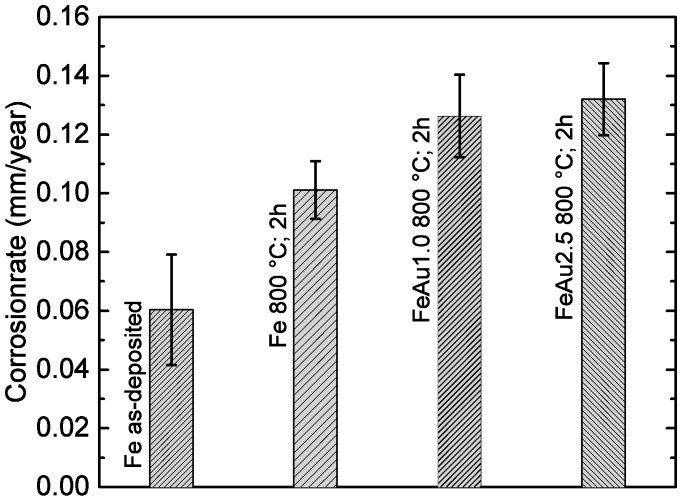
Mean corrosion rate of Fe, FeAu1.0 and FeAu2.5 samples. Using Equation (1), the corrosion rates were calculated from the corrosion current density *j_c_*, determined linear polarization in HBSS (pH 7.4; 37 °C) and Tafel extrapolation method.

**Figure 6:**
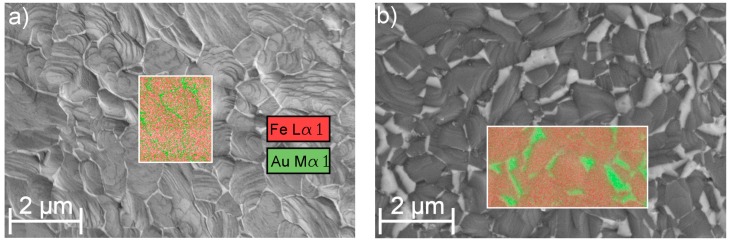
SEM surface image and EDX elemental mappings of the Fe-L-α and Au-M-α lines for (**a**) a FeAu1.0 and (**b**) FeAu2.5 sample annealed at 800 °C, 2 h. In the SEM image Au precipitates appearing bright due to the Z-contrast.

The results of the electrochemical and immersion tests are given in Figure 4 and Figure 5. In Figure 6a, the SEM image and EDX mapping is presented, showing the fine, distributed Au precipitates on the surfaces of a FeAu1.0 and a FeAu2.5 sample annealed at 800 °C for two hours. These samples show a clear difference in the amount of the precipitates even visible in Figure 3b–e, which preferred segregation along the grain boundaries. The difference of the corrosion rate between the as-deposited and the annealed Fe samples found in the electrochemical tests is discussed in a previous work [21] and is related to grain coarsening. However, the acceleration of the degradation rate for the annealed pure Fe was only observed for short immersion times (two days) and lost significance with longer immersion times. Thus, the influence of grain coarsening on the corrosion rate seems to be rather small, and is explained by the easier passive oxide formation on fine-grained surfaces due to an easier diffusion along grain boundaries [29]. Since in a fine-grained structure the grain boundary/grain ratio is higher, the material is able to form a stable passive oxide more efficiently. Both methods show an accelerated corrosion independent from the Au content of the Fe-Au samples compared to the as-deposited Fe samples and even the annealed Fe. While the difference of the degradation rate compared to pure Fe is significant in the electrochemical test as well as in the immersion test, the influence of different Au contents seems to be less distinct. However, at long immersion times (12 days) an influence of the Au content becomes more pronounced. The results are in good agreement with other studies [11,13] where the corrosion rate of the iron was found to be higher by implementing noble Pd, Pt, Ag and Au precipitates using powder metallurgy. 

### 3.2. Corrosion Measurements 

Au is one of the most noble elements in the galvanic series, and with its high standard potential of (*E*^0^_Au_ = 1.69 V) [30] it is much more noble than iron (*E*^0^_Fe_ = −0.44 V). It is well known [23] that in direct contact with a corroding metal, both metals form an metal- inert metal coupleand hence, a three-phase boundary at the surface (Fe/Au/electrolyte), where the Au acts as a cathode while and the iron as an anode. Thus, the Fe matrix and the Au precipitates form several micro-galvanic elements, leading to an accelerated average corrosion rate of the iron. The faster corrosion rate of the FeAu2.5 samples at 12 days of immersion time is explained by the larger cathode surface. This increased surface area promotes the reduction reaction since the reduction current has to be equal to the anodic current. As a consequence, the anodic metal dissolution has to increase. 

However, since the difference of the Au amount and thus the Fe/Au surface ratio (Figure 6) is rather small, the acceleration of the anodic Fe dissolution is only substantial in long-term immersion. The results proved that sputtering of Fe-Au multilayer systems is feasible to implement Au precipitates which accelerate the corrosion rate compared to the pure reference material. Since the corrosion reaction strongly depends on a number of parameters, e.g., pH value, gas concentrations, electrolyte flow, sample surface, impurity concentrations, cell and protein content of the electrolyte, further research has to show, to what extent the presented concept is valid under in vivo conditions.

## 4. Conclusions 

It is shown that the previously presented approach [12] of depositing magnetron-sputtered Fe-Au multilayer films and post-deposition heat treatments is feasible to tailor the mechanical properties and accelerate the corrosion speed of iron. While scaling with the Au amount, the strength and degradation speed is increased, the material loses ductility. With regard to the intended use as a biodegradable implant material, the amount of Au should be chosen as small as possible in order to minimize the number of Au particles remaining in the body. Thus, the FeAu1.0 samples showed the best compromise between low Au content, increased strength, sufficient ductility and accelerated degradation rate. However, it is very important to evaluate also the in vivo behavior to understand how the residual Au particles interact with a living organism. 

Sputtering allows the fabrication of alreadyfiligree-patterned Fe-Au foil with enhanced mechanical and degradation properties. Due to the very good process control, even the fabrication of devices with a gradient of the precipitation density could be realized. This would allow the fabrication of the ideal biodegradable implant where the outer part degrades initially rather slowly, while the core degrades faster and is able to keep its mechanical integrity longer due to its high strength in the core.

## Figures and Tables

**Table 1:** Sputtering parameters.

Element	Power (W)	Pressure (mbar)	Ar Gas Flow (sccm)	Sputtering Rate (nm/s)
Cu	1000 (DC *)	2.3 × 10^−3^	25	4.1
Fe	600 (RF #)	2.3 × 10^−3^	35	0.6
Au	200 (DC *)	2.3 × 10^−3^	35	1.0

* Direct current; # Radio frequency.

**Table 2:** Average grain size of the different samples annealed at 800 °C, 2 h. The values are determined from SEM surface images.

Sample	Average Grain Size (µm)
Fe [21]	3.01 ± 1.14
FeAu0.3	1.34 ± 0.91
FeAu1.0	1.23 ± 0.82
FeAu2.5	1.25 ± 0.74
